# Improvement of Cutaneous Anaplastic Large Cell Lymphoma by Brentuximab Vedotin Monotherapy

**DOI:** 10.4274/tjh.2017.0448

**Published:** 2018-05-25

**Authors:** Takashi Onaka, Tomoya Kitagawa, Chika Kawakami, Akihito Yonezawa

**Affiliations:** 1 Kokura Memorial Hospital, Clinic of Hematology, Kitakyushu, Fukuoka, Japan; 2 University of Occupational and Environmental, Department of Dermatology, Fukuoka, Japan

**Keywords:** Brentuximab vedotin, Cutaneous ALCL

Brentuximab vedotin (BV) is an antibody-drug conjugate composed of a CD30-directed monoclonal antibody and monomethyl auristatin E [1]. BV monotherapy showed good response rates for cases of refractory and relapsed anaplastic large cell lymphoma (ALCL), but only a few case reportsare available for cutaneous localized ALCL (cALCL). We herein report the treatment with BV of relapsed cALCL with an excellent response. An 82-year-old female with relapsed cALCL had generalized erythema accompanied by desquamation and could not extend her fingers enough (Figure 1), with no lymph node lesions. Due to the previous treatment with radiation, steroid ointment, and systemic chemotherapy, we chose BV monotherapy for her, dosing at 1.8 mg/kg every 21 days. After the third infusion, her generalized erythemaand her finger movement were improved (Figure 2). She did not have any severe adverse effects or infusion reaction except for hematologic toxicity (leukocytopenia). She has finished 6 courses of BV infusion and maintained remission of skin lesions. There are several reports that showed the effectiveness of BV treatment for cALCL [2,3], but the optimal treatment interval and cycles, and the necessity of maintenance therapy by using BV, are unclear. Further studies are needed to evaluate BV treatment in cases of cALCL.

## Figures and Tables

**Figure 1:**
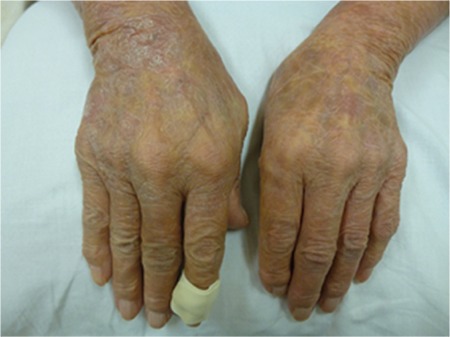
Generalized erythema accompanied by desquamation before treatment with brentuximab vedotin

**Figure 2:**
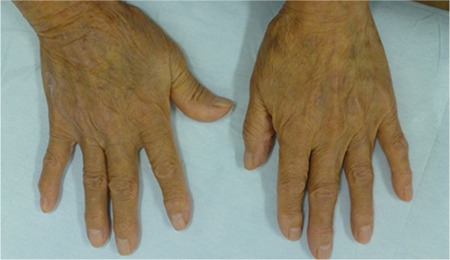
Improvement of skin erythema accompanied by desquamation after 4 cycles of brentuximab vedotin
